# Quantification of Cheese Yield Reduction in Manufacturing Parmigiano Reggiano from Milk with Non-Compliant Somatic Cells Count

**DOI:** 10.3390/foods9020212

**Published:** 2020-02-18

**Authors:** Piero Franceschi, Michele Faccia, Massimo Malacarne, Paolo Formaggioni, Andrea Summer

**Affiliations:** 1 Department of Veterinary Science, University of Parma, Via del Taglio 10, I-43126 Parma, Italy; piero.franceschi@unipr.it (P.F.); andrea.summer@unipr.it (A.S.); 2 Department of Soil, Plant and Food Sciences, University of Bari. Via Amendola 165/A, 70125 Bari, Italy; michele.faccia@uniba.it

**Keywords:** Parmigiano Reggiano cheese, somatic cells, milk composition, cheese yield, cheesemaking losses

## Abstract

The mammary gland inflammation process is responsible for an increased number of somatic cells in milk, and transfers into the milk of some blood components; this causes alterations in the chemical composition and physico-chemical properties of milk. For this reason, somatic cell count (SCC) is one of the most important parameters of milk quality; therefore, European Union (EU) Regulation no 853/2004 has stated that it must not exceed the limit value of 400,000 cells/mL. The research aimed to compare chemical composition, cheese yield, and cheesemaking losses of two groups of vat milks used for Parmigiano Reggiano production, characterized by different SCC levels. During two years, ten cheesemaking trials were performed in ten different cheese factories. In each trial, two cheesemaking processes were conducted in parallel: one with low SCC milk (below 400,000 cells/mL; Low Cell Count (LCC)) and the other with high SCC milk (400,000–1,000,000 cells/mL; High Cell Count (HCC)). For each trial, vat milk and cooked whey were analyzed; after 24 months of ripening, cheeses were weighed to calculate cheese yield. The HCC group had lower casein content (2.43 vs. 2.57 g/100 g; *p* ≤ 0.05) and number (77.03% vs. 77.80%; *p* ≤ 0.05), lower phosphorus (88.37 vs. 92.46 mg/100g; *p* ≤ 0.05) and titratable acidity (3.16 vs. 3.34 °SH/50 mL; *p* ≤ 0.05) compared to LCC. However, chloride (111.88 vs. 104.12 mg/100 g; *p* ≤ 0.05) and pH (6.77 vs. 6.71; *p* ≤ 0.05) were higher. Fat losses during cheesemaking were higher (20.16 vs. 16.13%). After 24 months of ripening, cheese yield was 8.79% lower for HCC milk than LCC (6.74 vs. 7.39 kg/100 kg; *p* ≤ 0.05).

## 1. Introduction

Mastitis is the inflammation of the mammary gland caused by bacterial infection. As a response to the inflammation, the number of macrophages, leucocytes, and polymorphonuclear cells strongly increases, causing a high level of the somatic cells in milk [1]. Somatic cell count (SCC) is one of the most important parameters of milk quality, both under the safety and technological point of views. As to safety, a high SCC level indicates poor hygienic quality and possible presence of pathogens. In order to protect the consumer’s health, the law (European Union (EU) Regulation no 853/2004) [2] has regulated this parameter for cow milk. In particular, the value (expressed as rolling geometric mean calculated over a period of three months, with at least one sampling per month) must not exceed the limit value of 400,000 cells/mL. Besides hygienic concerns, a high SCC level negatively influences the technological properties of milk, and both the coagulation process and the chemical-sensory characteristics of the cheeses tend to worsen. Several authors have investigated the causes of technological worsening, and they can be summarized in increased levels of casein passing into the soluble phase, greater proteolytic activity, and modifications of the balance of mineral salts [3,4,5].

An important issue connected with the application of Regulation 853/2004 [2] is that the limit of 400,000 cells/mL is calculated over a three-month period. During this period, the risk of milk not complying the legal limit that reaches the dairy transformation, does exist. Summer et al. [6] reported that 10.71% of the samples collected from free stalls in the Parmigiano Reggiano production area (Italy), exceeded the legal limit of milk production from 2006 to 2008. This was confirmed by data of the Lombardia and Emilia Romagna Experimental Zootechnical Institute (IZSLER, Italy). According to these results, about 10% of milk production in 2018 in these areas exceeded the 400,000 cells/mL limit [7]. These milks are mainly concentrated in the summer period [6], when the cows are subject to stress due to the hot and humid climate typical of the Po valley plain [8].

Two of the most produced cheeses in Lombardy and Emilia-Romagna regions are Grana Padano and Parmigiano Reggiano. Both have been recognized as EU Protected Designation of Origin (PDO) products [9] and are very similar from a chemical, technological, and nutritional point of view [10], being hard cooked cheeses manufactured by partially skimmed raw milk, added with autochthonous starter culture. According to the official protocol, partially skimmed milk from evening milking is merged with the full cream morning milk (about 50:50 *v*/*v*), giving rise to “vat milk”. Previous studies conducted on Parmigiano Reggiano reported wide variations of the composition of the vat milk throughout the year, and a significant decrease of the cheese yield when the milk somatic cells count exceeded 300,000 cells/mL [11,12]. Considering the importance of cheese yield on the economic efficiency of Parmigiano Reggiano cheesemaking, a specific study aiming to quantify the cheese yield capacity of milk with different levels of somatic cells is highly required. Such information could give a useful contribution to quantify the right remuneration of milk, motivating the breeders to make investments for reducing the somatic cells content. The aim of the present research is to compare chemical composition, cheese yield, and cheesemaking losses (during manufacturing to Parmigiano Reggiano) of milk with non-law-complying and law-complying SCC.

## 2. Materials and Methods

### 2.1. Parmigiano Reggiano Cheesemaking Process

Cheeses were produced according to the official protocol of Parmigiano Reggiano PDO cheese. Although the protocol does not include breed restrictions and allows commingling milk coming from different farms, the milk used in the experimentations was collected only from Italian Friesian cattle herds. Moreover, each cheesemaking trial was performed using milk derived from a single herd.

Vat milk was obtained by commingling the partially skimmed evening milk and the full cream morning milk. In brief, the whole milk of evening milking (WE-milk) was collected from the farm and transported to the cheese factory where it was placed into the creaming tank. Overnight, the cream was separated naturally from the milk and was collected. The morning after, the partially skimmed milk was obtained (fat content about 1.5 g 100 g^−1^), extracted from the bottom of the tank, and transferred into the cheesemaking vat; the same amount of the whole milk that was obtained by the morning milking of the same herd was added. The final merged milk was lower in fat, containing about 2.6 g 100 g^−1^ fat, with a fat to casein ratio of about 1–1.1. It is called vat milk (V-milk) and, before undergoing the cheesemaking process, is added with natural whey starter culture (2.5–3 liters 100 kg^−1^). The whey starter culture was obtained by spontaneous acidification of cooked whey (C-whey), deriving from the cheesemaking of the previous day. The inoculated vat milk was heated to 33 °C and clotted with 2.5–3 g 100 kg^−1^ of commercial calf rennet (1:120,000 strength). After 10–12 min, coagulation occurred; the curd was then cut into small granules (having approximately the size of a rice grain), and heated by increasing temperature to 55 °C. After cooking, the small curd particles were left to deposit at the bottom of the vat by decantation, and a whole curd mass was formed. During this time, the temperature remained approximately 53–55 °C. The curd mass was then removed from the vat, divided into two parts, and placed into molds for two day. They were periodically turned over to promote syneresis and whey draining. The cheese wheels were then salted in brine for a period of 20 days before entering the ripening room, where they remained for at least 24 months.

### 2.2. Experimental Design

Over a period of two years, ten comparative cheesemaking trials were performed in ten different cheese factories located in the Parmigiano Reggiano production area. In each trial, two cheesemaking processes were performed in parallel: one using V-milk coded as Low Cell Count (LCC) and the other using V-milk coded as High Cell Count (HCC). The classification LCC or HCC is based on SCC of the whole evening milk: LCC contained less than 400,000 cells/mL, whereas HCC contained more than 400,000 cells/mL, but less than 1,000,000 cells/mL.

### 2.3. Analytical Methods

In each cheesemaking trial, the following type of samples were collected: WE-milk taken from the tank of the farm; V-milk, taken from the vat before the addition of the natural whey starter; C-whey, taken from the vat after the extraction of the cheese, and stirred for 5 min. Sodium merthiolate (0.02 g/100 mL, as preservative) was added to all samples, which were cooled to 5 °C, and immediately transported to the laboratory for analyses.

The following tests were performed: somatic cells on WE- and V-milk using the fluoro-opto-electronic method with Fossomatic (Foss Electric, Hillerød, Denmark) [13]; fat content on V-milk by mid-infrared spectroscopy [14] with Milko-Scan FT6000 (Foss Electric); fat content of C-whey was determined by the volumetric Gerber method [15].

Total N (TN), non-casein N (NCN), and non-protein N (NPN) on V-milk, acid whey at pH 4.6 and Trichloroacetic acid (TCA 120 g/L; Carlo Erba Reagents, Milan, Italy) filtered whey, respectively, were determined by the Kjeldahl method, which was performed using a DK6 Digestor and UDK126A Distiller (VELP Scientifica, Usmate, Italy), according to the Association of Official Analytical Chemists (AOAC) standards [16,17,18]. From these analyses, crude protein (TN × 6.38/1000), casein ((TN-NCN) × 6.38/1000), casein number ((TN-NCN) × 100/TN), NPN × 6.38 (NPN × 6.38/1000), true protein ((TN-NPN) × 6.38/100) were calculated, as described by Summer et al. [12]. TN was also determined on C-whey, also by Kjeldahl [12]. 

The content of phosphorus was measured on V-milk and C-whey by colorimetric method [19] and those of calcium and magnesium by atomic absorption spectrometry (AAS Perkin-Elmer 1100 B, Waltham, MA, USA), as reported by Malacarne et al. [19]; chloride were determined on V-milk by argentometric Volhard method [20].

The pH was measured on V-milk with potentiometer Crison (Crison Instruments, E-08328 Barcelona, Spain), and titratable acidity by titration with sodium hydroxide solution (0.25 N) of 50 mL of V-milk using as indicator 2 mL of phenolphthalein in ethanol (20 g/L; Carlo Erba Reagents), according to the Soxhlet-Henkel method [12]. 

Somatic cell score (SCS = (Log_2_(SCC/100) + 3) [21] and fat-to-casein ratio values were calculated on WE-milk and V-milk, respectively.

For yield calculation, in each cheesemaking, V-milk was weighed directly in the vat, before the addition of the starter. Both the two cheese wheels resulting from each cheesemaking were weighed at 24 months of ripening; the moisture content of the cheese after drying at 102 °C was also determined [22].

The actual cheese yield (ACY; kg of cheese /100 kg of milk) was calculated as follows:

ACY = cw × 100/mw,

where: ACY = actual cheese yield; cw = cheese weight, expressed in kg; mw = milk weight, also expressed in kg.

The adjusted dry cheese yield (ADY; kg of cheese /100 kg of milk) was calculated, according to the following formula:

ADY = ACY × (100 − CMC)/100,

where: ADY = adjusted yield; CMC = cheese moisture content.

Finally, the estimated cheesemaking losses (ECL) values of protein, casein, fat, calcium, phosphorus, and magnesium were calculated as follows:

ECL = (C-whey) × 100/(V-milk),

where ECL is expressed as percentage; (C-whey) = concentration in whey, expressed as g/100 g (mg/100 g for Ca, P, Mg); (V-milk) = concentration in milk, expressed as g/100 g (mg/100 g for Ca, P, Mg).

### 2.4. Statistical Analysis

The significance of the differences between classes (LCC and HCC) was tested by analysis of variance, using the general linear model procedure of SPSS (IBM SPSS Statics 24, Armonk, NY, USA), according to the following univariate model:

Y_ijk_ = µ + C_i_ + T_j_ + ε_ijk_,

where: Y_ijk_ = dependent variable; µ = overall mean; C_i_ = effect of milk somatic cell class (LCC or HCC) (i = 1, 2); T_j_ = effect of trial (j = 1, ….10); ε_ijk_ = residual error. The significance of the differences was tested by least significant differences method.

Data were also processed by the Pearson product moment correlation coefficient, to measure the degree of the linear relationship between the somatic cell content of the whole evening milk (expressed as somatic cell score) and cheese yield, and between the vat milk chemical characteristics and cheese yield.

## 3. Results and Discussion

### 3.1. Chemical Composition and Physico-Chemical Properties of Vat Milk

The average somatic cell count in LCC WE-milk was 233,000 cells/mL (*minimum* 122,000; *maximum* 341,000 cells/mL) and 538,000 cells/mL (*minimum* 407,000; *maximum* 886,000 cells/mL) in HCC WE-milk. The fat and protein contents were 3.70 and 3.61 g/100 g, and 3.18 and 3.06 g/100 g, respectively for LCC and HCC-milk.

Chemical composition, physicochemical properties, and somatic cells count of LCC and HCC vat milk are described in Table 1. Casein, casein number, phosphorus, chloride, titratable acidity, and pH showed different values, with *p* ≤ 0.05.

The HCC V-milk, in comparison with the LCC V-milk, was characterized by lower casein (2.43 vs. 2.57 g/100 g; *p* ≤ 0.05) and phosphorus (88.37 vs. 92.46 mg/100 g; *p* ≤ 0.05) contents, lower casein number value (77.03 vs. 77.80%; *p* ≤ 0.05) and higher chloride content (111.88 vs. 104.12 mg/100 g; *p* ≤ 0.05). Moreover, titratable acidity was lower (3.16 vs. 3.34 °SH/50 mL; *p* ≤ 0.05) and the pH value was higher (6.77 vs. 6.71 units; *p* ≤ 0.05). Overall, the composition profile was less favorable for cheesemaking. In fact, cheese yield strictly depends on fat and casein contents [23], and pH, acidity, and mineral contents of the milk are closely linked to the rennet-coagulation properties [24].

The results are in accordance with those reported in the literature and collected in a review by Le Maréchal et al. [1]. More specifically, Franceschi et al. [11], in research performed on 248 Parmigiano Reggiano vat milk samples (202 with less than 400,000 cells/mL and 48 with more than 400,000 cells/mL), reported lower casein content (2.43 vs. 2.47 g/100 g *p* ≤ 0.05), casein number (76.78 vs. 77.42 *p* ≤ 0.05), and titratable acidity (3.24 vs. 3.29 °SH/50 mL *p* ≤ 0.05) for milk with more than 400,000 cells/mL than milk with less than 400,000. Summer et al. [25] observed, in a study conducted on 26 single quarter milk samples (13 with less and 13 with more than 400,000 cells/mL), a lower phosphorous content (87.76 vs. 89.71 mg/100 g *p* ≤ 0.05) and a higher chloride content (135.17 vs. 99.43 mg/100 g *p* ≤ 0.01).

All these changes in HCC milk are due to an increase of the concentration of whey protein [26,27] and sodium chloride [25], which come directly from blood [27], and a decrease of phosphorus [12,25] and casein [10], caused by decreased mammary activity and an increase of proteolytic enzymatic activity [26,28]. Being that titratable acidity and pH were highly correlated with the contents of casein and phosphorus, the significant lower and higher values of these two parameters, respectively, in HCC V-milk are explained.

### 3.2. Cheese Yield and Cheesemaking Losses of Vat Milk

**Figure 1:**
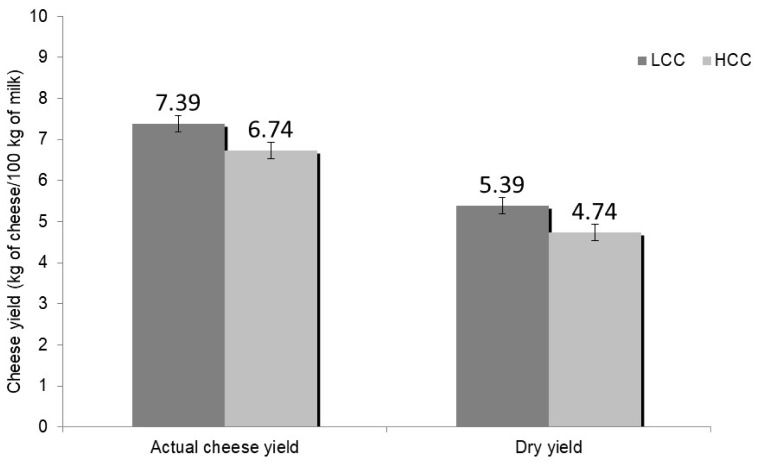
Cheese yield at 24 months ripening from milk with less than 400,000 (Low Cell Count (LCC)) and more than 400,000 cells/mL (High Cell Count (HCC)) (least square mean values). Classification was based on SCC of the whole evening milk. For both, LCC and HCC means differ with a *p*-value ≤ 0.05.

The least square mean values of cheese yield are shown in Table 2. Both actual cheese yields at 24 months of ripening and dry yield at 24 months of ripening were significantly lower in HCC cheesemaking than LCC (Figure 1).

In the literature, there is a general consensus about the negative relationships between somatic cells of milk and its cheese yield capacity. In hard Cheddar cheese production, a decrease of the yield was reported for milk with more than 100,000 cells/mL [4] and more than 300,000 cells/mL [29]. Similarly, in cottage (a soft cheese) production, Klei et al. [5] found that cheese yield efficiency was 4.34% lower for high somatic cell milk (mean value 872,000 cells/mL) than for low somatic cell milk (83,000 cells/mL). In contrast, Mazal et al. [30] observed no significant differences in Prato cheese yield when comparing milk with less than 200,000 cells/mL and more than 600,000 cells/mL (10.4 vs. 9.2 kg/100 kg, respectively). Such different results could depend on the low number of comparison made (only three comparative trials) and differences in cheesemaking technologies between Prato cheese (a soft cheese made with pasteurized milk) and hard cheeses. For Parmigiano Reggiano, the decrease of yield measured 24 h after the extraction of the curd started at 300,000 cells/mL [12]. The difference of the mean value of the 24-month cheese yield between the LCC milk and HCC was 0.65 kg/100 kg of processed milk, corresponding to a difference of 9.64%.

The difference in cheese yield were not caused by differences in water retention (the moisture of the cheeses was the same); this was confirmed by the calculation of the adjusted dry yield. In effect, cheese yield is directly proportional to casein and fat content [23]; moreover, the worsening of rennet coagulation properties [31], linked to the decrease in phosphorus and calcium, and to the reduction of the casein content itself, leads to an increase in the loss of fat in the whey.

**Figure 2:**
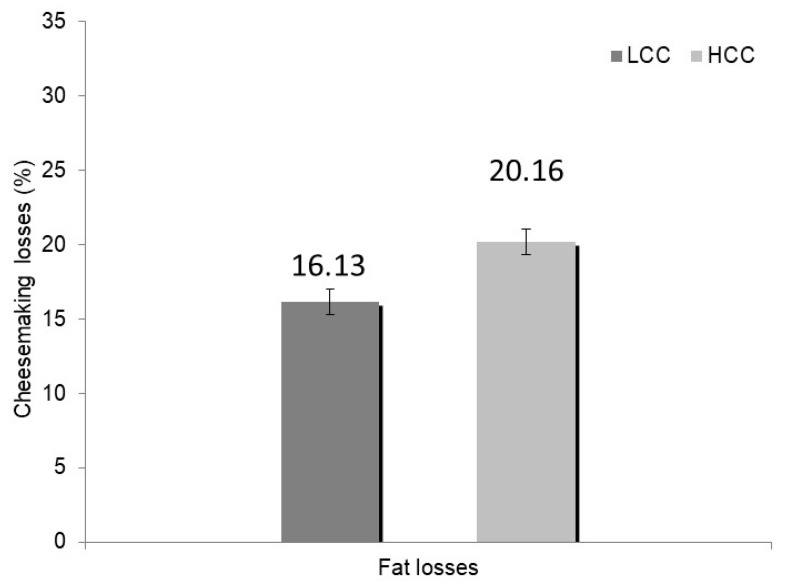
Cheesemaking fat losses of milk with less than 400,000 (LCC) and more than 400,000 cells/mL (HCC) (least square mean values). Classification was based on somatic cell count (SCC) of the whole evening milk. LCC and HCC means differ with a *p*-value ≤ 0.01.

In Table 2, fat, protein, and main minerals estimated cheesemaking losses are also shown. Only for fat losses, different average values (*p* ≤ 0.01) were observed between LCC and HCC V-milks, with higher losses in HCC-cheesemaking than in LCC (Figure 2). In a recent paper, Franceschi et al. [31] reported that the average fat loss in Parmigiano Reggiano, calculated on 288 cheesemaking trials, was 16.93%. In the present study, the fat loss in LCC-cheesemaking was in agreement with this value, while fat loss of the HCC-cheesemaking is much higher than in Franceschi et al. [31]. Poor efficiency of HCC curd in retaining fat into the casein reticulum may be both related to undesired structural modification of the curd and/or loss of integrity of the fat globule, resulting from the increased activities of plasmin and lipases in milk collected from infected-glands [4,32]. This observation was confirmed by the positive and significant correlation found between milk casein content and fat losses and between milk fat to casein ratio and fat losses (Table 3).

Pearson product moment correlation coefficients between WE-milk somatic cell content, cheese yield and cheesemaking losses, and between V-milk characteristics, cheese yield, and cheesemaking losses, are shown in Table 3.

A negative and significant correlation was observed between SCC (measured on the corresponding WE-milk before natural creaming) and V-milk cheese yield at 24 months ripening. Conversely, V-milk cheese yields were positively correlated with contents of protein, casein, and fat. Formaggioni et al. [23] reported, for their prediction formulas of the estimated cheese yield for hard cooked cheese types, a similar correlation between Parmigiano Reggiano cheese yield, and the content of milk crude protein, casein, and fat constituents. Furthermore, the same positive correlations are reported in other cheese typologies, as Grana Padano [33] and Saint-Nectaire cheeses [34].

## 4. Conclusions

In conclusion, the present study allowed quantifying the negative impact of milk somatic cell content on the cheese yield in manufacturing Parmigiano Reggiano cheese. The decrease of the yield both derives from the less favorable chemical composition of milk and from reduced efficiency of the coagulum to retain the fat fraction. It is very important to know that, even though Regulation (EC) no 853/2004 [2] indirectly allows to submit to cheesemaking milk with more than 400,000 cells/mL, due to the limited number of mandatory analyses, this must be avoided. The economic impact of the connected reduction of the cheese yield is not negligible, and could compromise profitability.

## Figures and Tables

**Table 1:** Chemical composition and physicochemical properties parameters of vat milk with less than 400,000 (Low Cell Count (LCC)) and more than 400,000 cells/mL (High Cell Count (HCC)) (least square mean values ± standard error).

Parameter	Unit of Measure	LCC ^1^ *n* ^2^ = 10	HCC ^1^ *n* ^2^ = 10	SE ^3^	*p* ^4^
Crude protein	g/100 g	3.30	3.16	0.06	NS
Casein	g/100 g	2.57	2.43	0.05	*
Casein number	%	77.80	77.03	0.31	*
NPN × 6.38	g/100 g	0.17	0.16	0.01	NS
True protein	g/100 g	3.13	3.00	0.06	NS
Fat	g/100 g	2.75	2.68	0.05	NS
Fat to casein ratio	Value	1.07	1.10	0.01	NS
Calcium	mg/100 g	114.75	114.17	1.54	NS
Phosphorus	mg/100 g	92.46	88.37	1.32	*
Magnesium	mg/100 g	11.12	10.70	0.25	NS
Chloride	mg/100 g	104.12	111.88	2.72	*
Titratable acidity	°SH/50 mL	3.34	3.16	0.04	*
pH	Value	6.71	6.77	0.02	*
Somatic cell count	10^3^ cells/mL	146	259	5	**

^1^ Classification was based on somatic cell count (SCC) of the whole evening milk; ^2^ Number of samples; ^3^ Standard error; ^4^ *p*-value: NS, *p* > 0.05; * *p* ≤ 0.05; ** *p* ≤ 0.01.

**Table 2:** Cheese yield and cheesemaking losses of vat milk with less than 400,000 (LCC) and more than 400,000 cells/mL (HCC) (least square mean values ± standard error).

Parameter	Unit of Measure	LCC ^1^ *n* ^2^ = 10	HCC ^1^ *n* ^2^ = 10	SE ^3^	*p* ^4^
Cheese yield:					
Actual cheese yield at 24 months	kg/100 kg	7.39	6.74	0.18	*
Dry yield at 24 months	kg/100 kg	5.19	4.74	0.20	*
Cheese characteristics:					
Moisture	g/100 g	29.84	29.78	0.28	NS
Estimated cheesemaking losses:					
Protein	%	26.59	26.92	0.28	NS
Casein	%	5.65	5.13	0.22	NS
Fat	%	16.13	20.16	0.87	**
Phosphorus	%	49.86	50.28	0.87	NS
Calcium	%	33.86	34.71	0.51	NS
Magnesium	%	76.92	77.36	1.69	NS

^1^ Classification was based on SCC of the whole evening milk; ^2^ Number of samples; ^3^ Standard error; ^4^ *p*-value: NS, *p* > 0.05; * *p* ≤ 0.05; ** *p* ≤ 0.01.

**Table 3:** Pearson correlation coefficient (*r*) between somatic cell content of whole evening milk, cheese yield and cheesemaking losses, and between the vat milk chemical parameters, cheese yield and cheesemaking losses.

	Cheese Yield ^1^		Protein Losses		Fat Losses	
	*r*	*p* ^2^	*r*	*p* ^2^	*r*	*p* ^2^
Somatic cells ^3^	−0.57	*	0.06	NS	0.34	NS
Crude protein	0.68	*	0.03	NS	−0.58	**
Casein	0.69	*	−0.20	NS	−0.64	**
Fat	0.60	*	−0.09	NS	−0.34	NS
Fat to casein ratio	−0.18	NS	0.15	NS	0.46	*

^1^ At 24 months ripening; ^2^ *p*-value: NS, *p* > 0.05; * *p* ≤ 0.05; ** *p* ≤ 0.01; ^3^ Expressed as somatic cell score (SCS).
